# The cost of ongoing war: escalation of continuous traumatic stress response over time and later functional, adjustment, and affective difficulties

**DOI:** 10.3389/fpsyt.2026.1891039

**Published:** 2026-08-14

**Authors:** Roy Aloni, Anna Harwood-Gross, Bar Lambez

**Affiliations:** 1 Department of Psychology, Ariel University, Ariel, Israel; 2 METIV Israel Psychotrauma Center, Herzog Medical Center, Jerusalem, Israel; 3 Department of Psychology, Ruppin Academic Center, Emek Hefer, Israel; 4 Loewenstein Hospital, Ra’anana, Israel

**Keywords:** adjustment, anxiety, continuous traumatic stress response, depression, functioning, Israel–Hamas war, longitudinal study

## Abstract

**Background:**

Continuous Traumatic Stress Response (CTSR) refers to psychological, emotional, and behavioral reactions that emerge under conditions of ongoing, realistic, and future-oriented threat. Recently, CTSR received empirical attention in areas affected by political armed conflict; studies have been cross-sectional. Therefore, there is limited understanding of CTSR dynamics over time.

**Methods:**

The current longitudinal study examined changes in CTSR during the Israel–Hamas war and its associations with functioning problems, maladjustment, and psychological burden (anxiety and depression). The study included 519 Israeli adults assessed during weeks 4–6 of the war (T1), and 264 participants during weeks 23–36 (T2). CTSR was evaluated at both waves, and war exposure, functioning problems, maladjustment, and psychological burden at follow-up. Latent change score models estimate within-person change in CTSR and the prospectively associaitons with follow-up outcomes.

**Results:**

Higher baseline CTSR and greater increases in CTSR were prospectively associated with greater functioning problems, maladjustment, and psychological burden at follow-up.

**Conclusions:**

CTSR may be a dynamic indicator of accumulating psychological risk. Monitoring changes in CTSR during prolonged conflict may help identify individuals at increased risk for later functional impairment, maladjustment, and emotional difficulties. CTSR-informed assessment may improve early identification, triage, and prevention efforts in populations exposed to continuing danger.

## 1 Introduction

Living under prolonged and realistic threat requires ongoing psychological, behavioral, and physiological adaptation. Unlike discrete traumatic events that can be processed after the threat has passed, continuous exposure keeps individuals in a constant state of anticipation. This can erode their adaptive capacity, especially when the threat is unpredictable and integrated into daily life. The cumulative effects of such threats align with allostatic load models, which explain how persistent activation and dysregulation of systems like the hypothalamic–pituitary–adrenal axis can lead to psychological distress and increased vulnerability to disease (1, 2). Thus, ongoing traumatic stress may be understood as a chronic state of living with constant danger.

The conceptualization of Continuous Traumatic Stress (CTS) refers to circumstances in which individuals or communities are exposed to repeated, ongoing, and realistic threats in daily life (3). Contemporary interpretations describe CTS as a distinct domain within the traumatic stress spectrum, marked by the ongoing alternation between everyday functioning and periods of active threat (4). This distinction matters because Post-traumatic Stress Disorder (PTSD) frameworks were built around past events that have ended and include four clusters of symptoms: intrusion symptoms, avoidance symptoms, hyperarousal symptoms, and changes in cognition and mood symptoms; while CTSR captures distress under ongoing danger (5, 6).

The present study was conducted in Israel following the Hamas-led attack of October 7, 2023, which marked the beginning of the Israel–Hamas war. The attack included large-scale rocket fire and infiltration into civilian communities in southern Israel by land, air, and sea, causing ~1200 deaths and kidnapping 251 hostages. Recent population-based work emphasized the complexity of exposure to mass-casualty conflict and terror stress following October 7, including exposure domains such as missile attacks, physical violence, evacuation, combat participation, hostage involvement, media exposure, and group-based marginalization, and showed that these domains were associated with PTSD, depression, anxiety, and somatic symptoms (7). A nationwide prospective study conducted shortly after the attack documented a broad and significant increase in PTSD, depression, and anxiety among Israeli adults, underscoring the scale of the psychological impact on the general population (8).

In the weeks and months that followed, Israeli civilians were exposed not only to the acute aftermath of the attack but also to continuing missile alerts, uncertainty regarding hostages, military mobilization, evacuation from border areas, and the possibility of escalation on multiple fronts. This context created a prolonged and evolving threat environment in which danger was not limited to a discrete past event but remained embedded in everyday life. Accordingly, the Israel-Hamas war provides a clinically relevant setting for examining responses to ongoing, realistic, and future-oriented threat.

Recent work by Goral et al. (5) has formalized the psychological reactions to CTS using the construct of continuous traumatic stress response (CTSR). The researchers developed and validated the CTSR scale among communities bordering the Gaza Strip in southern Israel that were exposed to ongoing security threats. Their findings showed that CTSR was moderately associated with PTSD symptoms but represented a related yet distinct construct, and that CTSR was strongly associated with distress and functional impairment. These findings suggest that CTSR captures a broader clinical profile than PTSD, including emotional exhaustion, perceived betrayal, helplessness, and reduced perceived safety.

A growing body of recent research has further demonstrated that CTSR is a context-sensitive construct. Regarding the Russo-Ukrainian war, Frankova et al. (9) adapted the CTSR scale for Ukrainian civilians and identified a revised three-factor structure: exhaustion, alienation, and helplessness. The Ukrainian version demonstrated acceptable reliability and convergent validity with depression, anxiety, perceived stress, resilience, and PTSD symptoms, while maintaining construct distinctiveness from PTSD. Extending this work to Ukrainian military personnel, Avramchuk et al. (10) identified a related but context-specific CTSR profile. Their findings suggested that CTSR among military personnel overlaps with depressive and burnout-like manifestations, yet remains empirically distinct from PTSD. Together, these studies suggest that CTSR may manifest differently across civilian and military populations and across sociopolitical contexts, underscoring the need for context-sensitive measurement and interpretation.

The relevance of CTSR has become increasingly evident in Israel following the Hamas-led attack of October 7, 2023, and the subsequent Israel-Hamas war. A recent study by Harwood-Gross et al. (11), conducted during weeks 4–6 of the war, found that direct exposure was not independently associated with CTSR levels. Rather, extreme media exposure, lower forward-focused coping, and lower system justification were associated with higher CTSR. These findings suggest that, during the early phase of continuous threat, CTSR may be shaped not only by objective exposure but also by subjective coping capacities, media exposure, and trust in broader societal systems (11). As this study was conducted during the early phase of the war, participants had experienced the initial traumatic events, but the cumulative consequences of repeated exposure to danger, sustained uncertainty, disruption of daily life, and the gradual depletion of personal and social coping resources (12, 13) may contribute to an escalation in CTSR over time. Nevertheless, stabilization or adaptation remained plausible individual responses; thus, the hypothesis concerned the average trajectory of CTSR across the sample rather than an expectation that all participants would exhibit increasing distress.

The potential consequences of CTSR are particularly relevant to psychopathology, functioning, and broader adjustment. Although CTSR is conceptually distinct from PTSD, empirical studies consistently show that it is associated with multiple domains of psychological distress and impairment. In the original validation study, CTSR scores were strongly correlated with distress and functional impairment, suggesting that CTSR is not merely a contextual stress reaction but is closely linked to everyday functioning and subjective impairment (5). Similarly, the Ukrainian civilian adaptation found positive associations between CTSR and symptoms of depression, anxiety, perceived stress, and PTSD symptoms, supporting the possibility that CTSR may reflect a broader vulnerability to psychological distress. In that study, CTSR was negatively associated with resilience, which was conceptualized as the perceived capacity to recover or “bounce back” from stress. This finding suggests that greater CTSR symptoms co-occurred with a lower perceived ability to recover from stress under conditions of prolonged war (9). In Ukrainian military personnel, CTSR was associated with depression, PTSD symptoms, and functional impairment, while its factor structure included helplessness–depression and psychosocial disruption, further indicating that continuous threat may be expressed not only through traumatic stress symptoms but also through affective burden and disruption in interpersonal and occupational functioning (10). Taken together, this literature provides a strong rationale for examining whether CTSR escalation is prospectively associated with subsequent psychopathology, functioning problems, and maladjustment.

Despite recent progress in scale development and cross-cultural validation, CTSR research remains limited, especially on how CTSR changes over time and whether change predicts later impairment. Moreover, there is less understanding of how CTSR over time can predict future psychopathology and functioning. This gap is especially important because CTSR is theoretically based on the cumulative burden of ongoing danger. If CTSR represents sustained adaptation to continuous threats, its clinical significance may not only lie in its severity at a single time point but also in its escalation with continued exposure.

The present study addresses this gap by examining longitudinal change in CTSR during the Israel-Hamas war and its prospective associations with later functioning and psychological adjustment. Participants were drawn from an initial sample of 519 Israeli adults assessed during weeks 4–6 of the war and reassessed several months later, during weeks 23–36. The final longitudinal sample included 264 participants. Using latent change score modeling, we examined whether baseline CTSR and within-person increases in CTSR associated with three clinically relevant outcome domains at follow-up: functioning problems, maladjustment, and affective psychopathology, namely anxiety and depressive symptoms. We hypothesized that (1) CTSR would increase over time, reflecting the cumulative burden of prolonged exposure to ongoing threat. (2) We further hypothesized that both higher baseline CTSR and greater increases in CTSR would prospectively associate with greater functioning problems, higher maladjustment, and elevated anxiety and depressive symptoms at follow-up, even after accounting for demographic characteristics and war exposure.

## 2 Methods

### 2.1 Participants and procedure

Participants were recruited from a sample of 519 people living in Israel during the Israel-Hamas war. The initial sample was recruited from social media, university mailing lists, and neighborhood WhatsApp groups, between October and November 2023 (weeks 4 to 6 after the Hamas attack on October 7, 2023). The current study was considered the second wave of a longitudinal study and was conducted between March and June 2024 (approximately weeks 23 to 36 after the Hamas attack on October 7, 2023). During this period of time, there were on average 629 home front emergency alerts sent per month (a siren sounded to indicate an incoming rocket and need to go to a safe space) compared to 7918 alerts in October 2023 and 1314 in November 2023 (during the first wave) (INSS, The 14). Emergency alerts are reported in real time on television and radio, and via push notifications, even if not in the relevant geographic region. Participants were contacted by telephone, and if they agreed, they were sent a WhatsApp link to an online survey and then reminded if they hadn’t completed it. The survey was hosted on the Qualtrics platform, and participants were given a unique identifying number (1-519) in their WhatsApp messages to link the two data sets while maintaining anonymity. Participants filled in contact details if they wanted to be included in a draw to win a 500 NIS voucher.

The current study included 264 of 519 participants who took part in the first wave (for more details, see 11). For the current wave, 312 participants responded to the study questionnaires, including 264 who completed more than 90% of the survey and 48 who completed less than 50% and were excluded from the sample. Among the 207 participants who did not answer the questionnaires, 125 did not respond to repeated telephone and email contact attempts, 60 initially expressed willingness to participate but ultimately did not complete the survey, 12 declined participation during the telephone contact, and 10 could not be reached because of an invalid telephone number and the absence of a valid email address.

The participants aged 19 to 77 (*M* = 40.89, *SD* = 11.80); the majority were females (87%), and most were parents (81%). Additionally, the sample was predominantly Jewish (98%). See **Table 1** for more sociodemographic information. The attrition rate of 49% of the initial sample is similar to that in other longitudinal studies (15) and, given the wartime setting, was considered acceptable. Independent-samples t-tests indicated that there were no significant differences between responders and non-responders in CTSR symptom levels (p=0.23), depression-anxiety levels (p=0.12), adjustment (p=0.34), or in functioning (p=0.11). The research was approved by the Ariel University ethics committee (AU-SOC-RA-20240316).

**Table 1:** Demographic characteristics of the sample (n=264).

Variable	*N*	%
Personal status		
Single	33	13%
Married	188	71%
In relationship	27	10%
Divorced/separated	10	4%
Widowed	3	1%
Other	3	1%
Residence location		
South	22	8%
Gaza border	2	1%
Center	158	60%
North	82	31%
Economic status		
Below average	20	8%
Average	162	61%
Above average	82	31%
Education		
No certification	4	2%
High school	19	7%
Undergraduate	87	33%
Masters	130	49%
PhD	24	9%

### 2.2 Measures

#### 2.2.1 Demographic variables

In addition to the demographic variables collected in wave 1 (age, sex, personal status, residence location, economic status, education, and religious affiliation), in wave 2, participants were asked about any change in these variables in the recent months, and in the relevant cases, the status was updated.

#### 2.2.2 War exposure

War exposure was assessed using an adapted version of the Mass, Multidimensional Armed-Conflict Trauma Measure (7), a recently developed instrument designed to capture the multidimensionality of exposure to mass-casualty armed conflict and terror. The original identifies seven exposure domains relevant to the October 7 events and their aftermath: missile attacks, violence, evacuation, hostage involvement, combat participation, intensive media exposure, and group-based marginalization. Participants were asked whether they were exposed to each domain (“1”) or not (“0”). The score was calculated as the sum of all their answers.

#### 2.2.3 Continuous traumatic stress response

The study’s outcome variable, CTSR, was measured by the CTSR scale (5). The CTSR questionnaire contains 11 statements that are divided into three factors: the exhaustion and detachment factor (e.g., “I feel unmotivated”), the anger and betrayal factor (e.g., “I have episodes of rage”), and the fear and helplessness factor (e.g., “I feel that I cannot protect those that depend on me”). The participant is asked to rate the statements on a Likert scale from 0 (not at all) to 3 (to a great extent), and the total score is calculated by summing item ratings. The higher the score, the more severe the CTSR symptoms. The questionnaire was validated in a comparative study between a sample of residents living adjacent to the Gaza Strip and a sample of residents of central Israel, who were not exposed to prolonged missile attacks (5), and was found to have discriminative validity in relation to symptoms on the Posttraumatic Diagnostic Scale (PDS-5). In the current study, we used the total score, with an internal consistency of α = .84.

#### 2.2.4 Depression and anxiety

Depression and anxiety symptoms were assessed using the Patient Health Questionnaire-4 (PHQ-4) is a validated ultra-brief screening instrument consisting of four items that measure core symptoms of anxiety and depression. The PHQ-4 comprises two subscales: the Generalized Anxiety Disorder-2 (GAD-2) and the Patient Health Questionnaire-2 (PHQ-2), each including two items rated on a 4-point Likert scale ranging from 0 (“not at all”) to 3 (“nearly every day”). The PHQ-4 has demonstrated good internal consistency, construct validity, and sensitivity to detecting clinically relevant anxiety and depressive symptoms across diverse populations (16, 17). In the current study, the internal consistency was α = .86 for the anxiety scale and α = .81 for the depression scale.

#### 2.2.5 Adjustment and functioning

Adjustment and functioning were assessed using the International Adjustment Disorder Questionnaire (IADQ), a self-report measure developed to operationalize diagnostic criteria for adjustment disorder according to the ICD-11 framework. The IADQ assesses exposure to a recent stressor and core symptom domains, including preoccupation with the stressor and failure to adapt, along with associated functional impairment. 6 items are rated on a Likert-type scale from “0” (not at all) to “4” (to a great extent), with higher scores indicating higher levels of maladjustment. The functional section included 3 items on social functioning, work and study functioning, and other aspects of functioning. The instrument has demonstrated good internal consistency and construct validity across diverse populations (18). In the current study, the Hebrew version of the IADQ was used, which has been validated in Israeli samples and has demonstrated robust psychometric properties comparable to those of the original version (19). The internal consistency was α = .88. In addition,

### 2.3 Data analysis strategy

**Figure 1:**
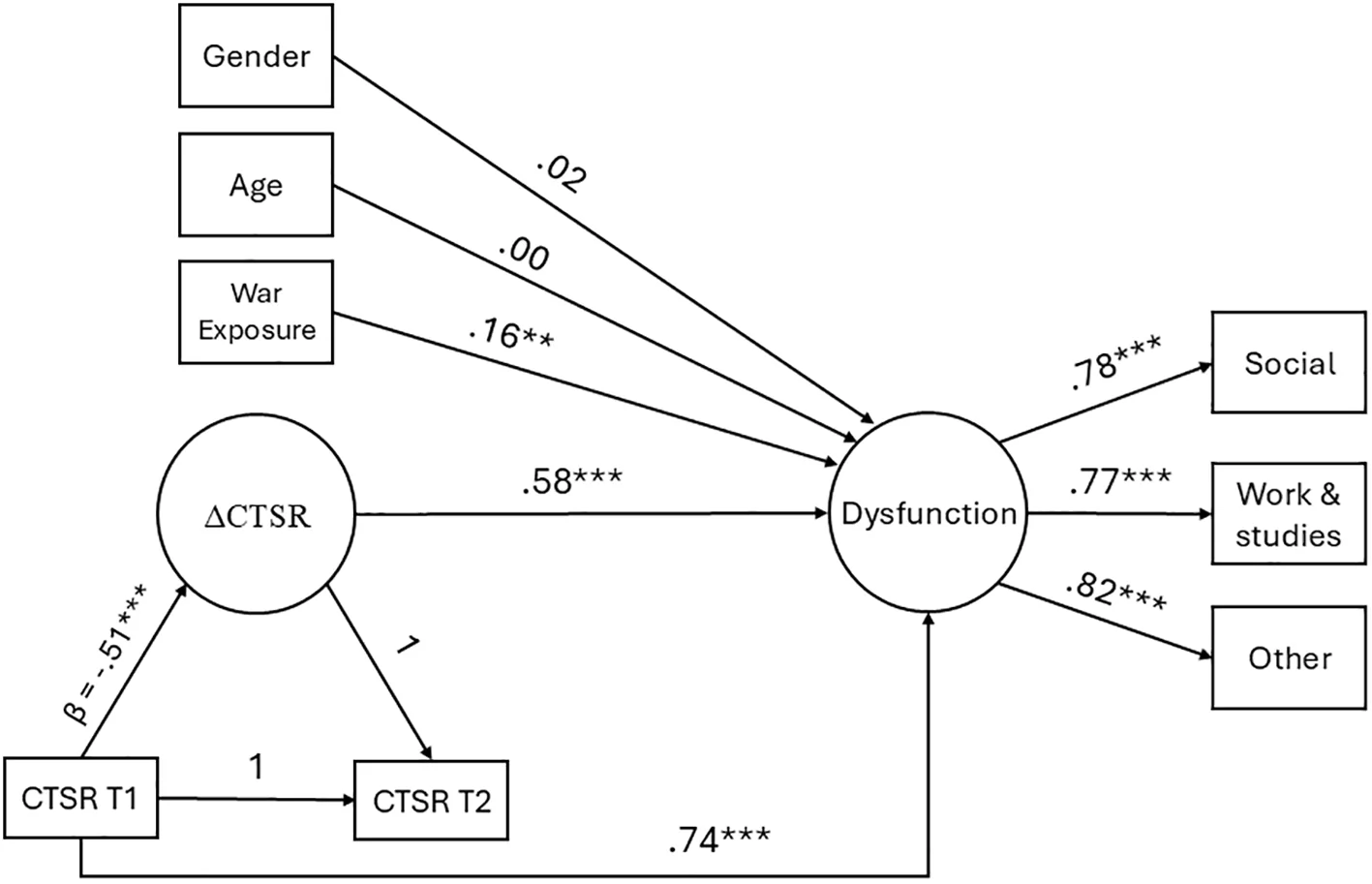
LCS model predicting functioning problems at T2. Free parameters are presented in standardized form. **p< .01, ***p<.001.

**Figure 2:**
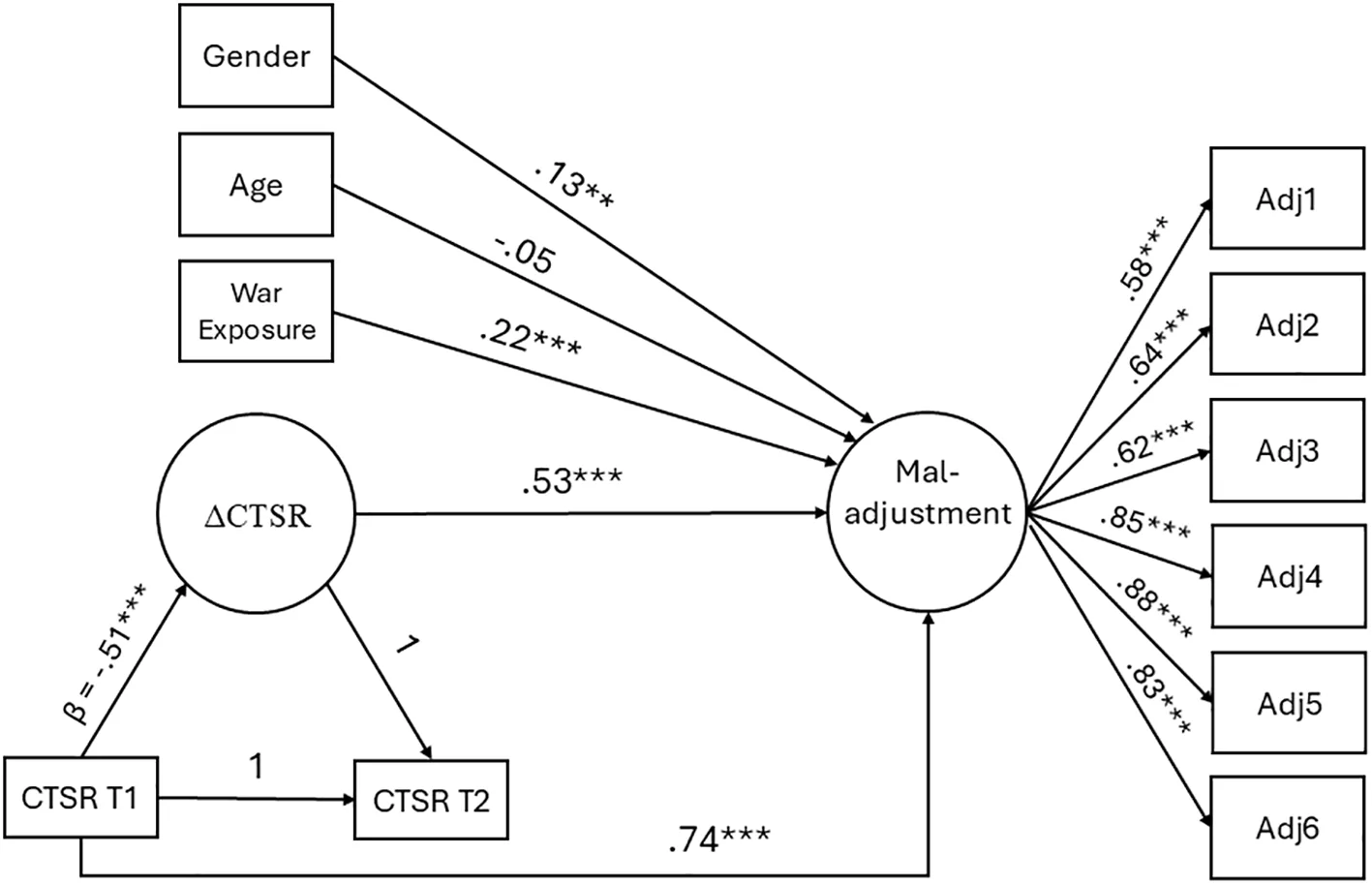
LCS model predicting maladjustment at T2. Free parameters are presented in standardized form. **p < .01, ***p < .001.

**Figure 3:**
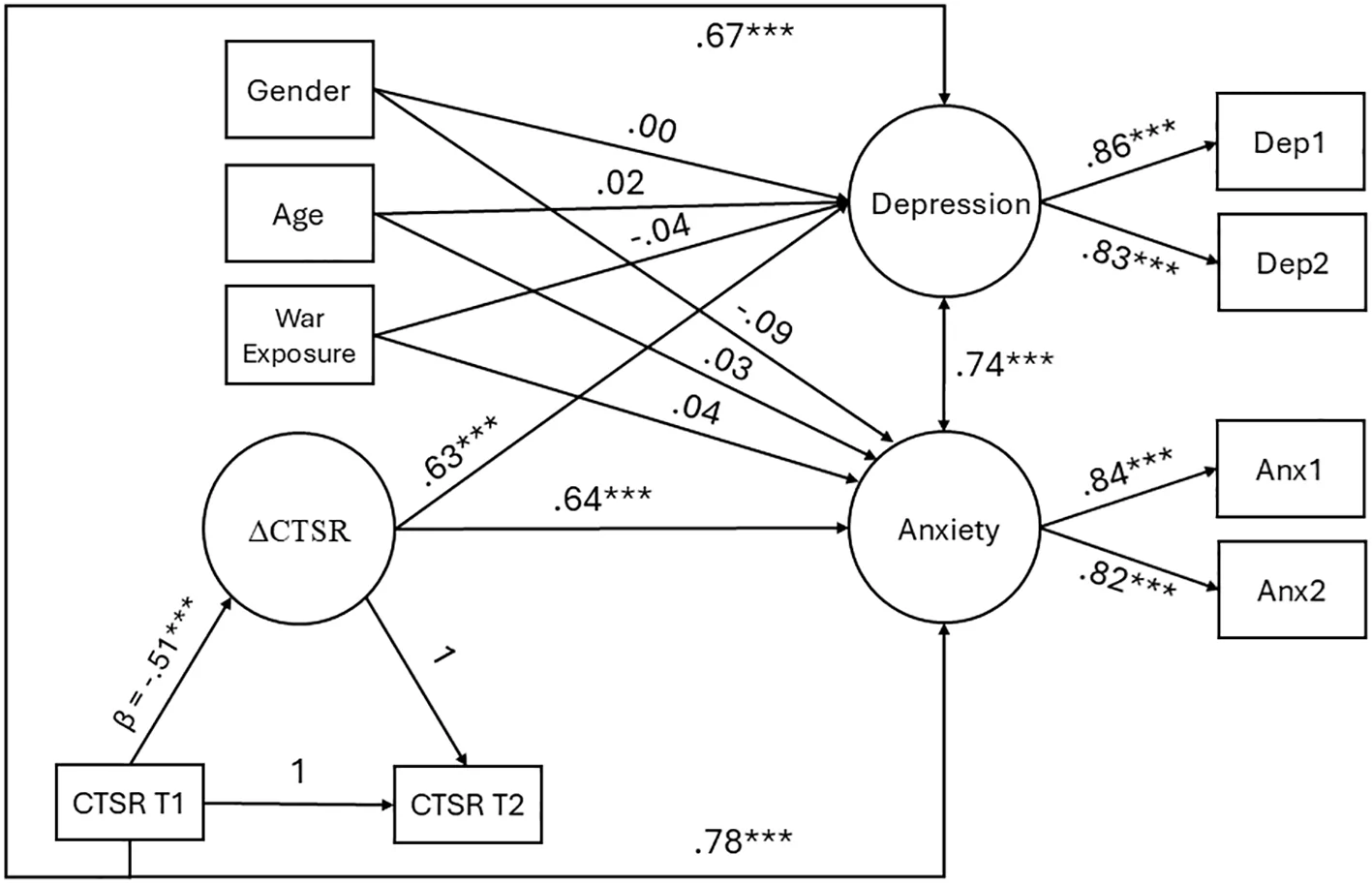
LCS model predicting anxiety and depression at T2. Free parameters are presented in standardized form. ***p < .001.

To examine mental health and functioning regarding changes in CTSR scores, we employed a structural equation modeling approach, specifying three latent change score (LCS) models (20) to examine functioning problems, maladjustment, and affective symptoms (depression and anxiety) at T2. In an LCS model, a latent change variable is estimated from the scores at T1 and T2, while accounting for the dependency between T1 scores and the degree of change from T1 to T2. In the first LCS model, latent change in CTSR scores was prospectively associated with a latent variable representing the shared variance of the three functioning items (see **Figure 1**). In the second LCS model, latent change in CTSR scores was associated with a latent variable representing the shared variance among the six adjustment items (see **Figure 2**). In the third LCS model, latent change in CTSR scores was associated with two latent variables, representing the shared variances of the anxiety and depression items of the PHQ-4, respectively (see **Figure 3**). By using a latent-variable approach, we could estimate the association between changes in CTSR scores and the dependent variables while partialling out measurement error in the dependent variables. In each model, we controlled for the CTSR score at T1, gender, age, and war exposure. All analyses were conducted using Mplus 8.8 (21). Missing data were handled using full information maximum likelihood (FIML) estimation. Model fit was examined by comparing approximate fit indices to commonly accepted benchmarks [Comparative Fit Index (CFI) >.95; root mean squared error of approximation (RMSEA) <.06; standardized root mean squared residual (SRMR) <.08; 22].

## 3 Results

### 3.1 Descriptive statistics and preliminary analyses

Zero-order correlations and descriptive statistics for all study variables are presented in **Table 2**. As expected, all measures of emotional distress were intercorrelated, and almost all, except CTSR at T1 and depression at T2, showed positive associations with war exposure. Preliminary LCS modeling revealed that CTSR scores increased significantly from T1 to T2 (ΔCTSR = 9.455, *SE* = 0.29, *t* = 32.44, *p* <.001, *d* = 1.99). Individuals with higher CTSR scores at T1 increased less from T1 to T2 (*B* = -0.422, *SE* = 0.044, *t* = -9.53, *p* <.001, β =-.506).

**Table 2:** Descriptive statistics and zero-order correlations between study variables.

Variables	1	2	3	4	5	6	7	8	9	10
1. CTSR T1										
2. CTSR T2	.63^***^									
3. Social dysfunction T2	.39^***^	.56^***^								
4. Work/school dysfunction T2	.37^***^	.50^***^	.61^***^							
5. Other dysfunction aspects T2	.35^***^	.58^***^	.63^***^	.63^***^						
6. Maladjustment T2	.44^***^	.63^***^	.52^***^	.52^***^	.59^***^					
7. Anxiety T2	.43^***^	.66^***^	.53^***^	.45^***^	.51^***^	.55^***^				
8. Depression T2	.32^***^	.59^***^	.42^***^	.37^***^	.44^***^	.45^***^	.70^***^			
9. War exposure T2	.13	.18^**^	.21^**^	.25^***^	.23^***^	.28^***^	.18^**^	.07		
10. Age	-.20^***^	-.07	.01	-.12^*^	-.05	.09	-.04	-.01	-.06	
*M*	12.45	21.91	2.66	2.59	2.95	18.95	3.99	3.95	3.85	40.78
*SD*	5.69	5.25	1.08	1.14	1.02	5.52	1.64	1.64	1.93	11.87
*N*	264	264	258	258	258	258	252	252	230	264

Maladjustment, anxiety, and depression are represented by sum scores.

*p < .05, **p < .01, ***p < .001.

### 3.2 LCS model examining functioning problems at T2

The results of the LCS model are displayed in **Table 3** and **Figure 1**. Higher CTSR scores at T1 were associated with more functioning problems at T2 (*B* = 0.109, *SE* = 0.011, *t* = 10.07, *p* <.001, β = .735). Additionally, a greater increase in CTSR scores from T1 to T2 was associated with more functioning problems at T2 (*B* = 0.102, *SE* = 0.012, *t* = 8.60, *p* <.001, β = .575). Finally, greater war exposure predicted more functioning problems at T2 (*B* = 0.071, *SE* = 0.024, *t* = 2.91, *p* = .004, β = .162). The model fit the data well (χ^2(14)^ = 28.15, *p* = .014; CFI = .975; RMSEA = .062; SRMR = .045) and explained 50.9% of the variance in functioning at T2.

**Table 3:** Maximum likelihood estimates for the LCS model predicting functioning problems at T2.

Statistical parameters	*B*	*SE*	*t*	*p*	β	95% CI[*B*]
ΔCTSR	0.102	0.012	8.60	<.001	.575	[0.079, 0.125]
CTSR T1	0.109	0.011	10.07	<.001	.735	[0.088, 0.130]
Age	0.000	0.004	0.01	.991	.001	[-0.007, 0.008]
Gender	0.037	0.132	0.28	.778	.015	[-0.222, 0.296]
Exposure to war	0.071	0.024	2.91	.004	.162	[0.023, 0.118]

Gender: 1 = Woman, 2 = Man. *R*^2^ = .509. Fit indices: χ^2(14)^ = 28.15, *p* = .014; CFI = .975; RMSEA = .062; SRMR = .045.

### 3.3 LCS model examining maladjustment at T2

The results of the LCS model are displayed in **Table 4** and **Figure 2**. Higher CTSR scores at T1 were associated with greater maladjustment at T2 (*B* = 0.073, *SE* = 0.009, *t* = 8.20, *p* <.001, β = .735). In addition, a greater increase in CTSR scores from T1 to T2 was associated with greater maladjustment at T2 (*B* = 0.063, *SE* = 0.009, *t* = 7.09, *p* <.001, β = .528). Finally, older age (*B* = 0.006, *SE* = 0.002, *t* = 2.57, *p* = .010, β = .134) and greater war exposure (*B* = 0.063, *SE* = 0.016, *t* = 3.88, *p* <.001, β = .218) predicted more maladjustment at T2. The model fit the data well (χ^2(34)^ = 51.67, *p* = .027; CFI = .986; RMSEA = .044; SRMR = .045) and explained 52.5% of the variance in maladjustment at T2.

**Table 4:** Maximum likelihood estimates for the LCS model predicting maladjustment at T2.

Statistical parameters	*B*	*SE*	*t*	*p*	β	95% CI[*B*]
ΔCTSR	0.063	0.009	7.09	<.001	.528	[0.045, 0.080]
CTSR T1	0.073	0.009	8.20	<.001	.735	[0.055, 0.090]
Age	0.006	0.002	2.57	.010	.134	[0.002, 0.011]
Gender	-0.089	0.082	-1.08	.280	-.054	[-0.250, 0.072]
Exposure to war	0.063	0.016	3.88	<.001	.218	[0.031, 0.095]

Gender: 1 = Woman, 2 = Man. *R*^2^ = .525. Fit indices: χ^2(34)^ = 51.67, *p* = .027; CFI = .986; RMSEA = .044; SRMR = .045.

### 3.4 LCS model examining anxiety and depression at T2

The results of the LCS model are displayed in **Table 5** and **Figure 3**. Higher CTSR scores at T1 were associated with greater anxiety (*B* = 0.097, *SE* = 0.009, *t* = 11.10, *p* <.001, β = .784) and depression (*B* = 0.091, *SE* = 0.009, *t* = 9.65, *p* <.001, β = .674) at T2. In addition, a greater increase in CTSR scores from T1 to T2 was associated with greater anxiety (*B* = 0.094, *SE* = 0.010, *t* = 9.59, *p* <.001, β = .635) and depression (*B* = 0.102, *SE* = 0.011, *t* = 9.53, *p* <.001, β = .633) at T2. Unlike in the prior two models, exposure to the war did not predict anxiety or depression. The model fit the data well by most fit indices (χ^2(15)^ = 38.09, *p* <.001; CFI = .972; RMSEA = .076; SRMR = .041) and explained 53.3% of the variance in anxiety and 41.4% of the variance in depression at T2.

**Table 5:** Maximum likelihood estimates for the LCS model predicting anxiety and depression at T2.

	*B*	*SE*	*t*	*p*	β	95% CI[*B*]
Anxiety						
ΔCTSR	0.094	0.010	9.59	<.001	.635	[0.075, 0.113]
CTSR T1	0.097	0.009	11.10	<.001	.784	[0.080, 0.114]
Age	0.002	0.003	0.54	.589	.030	[-0.005, 0.008]
Gender	-0.191	0.109	-1.74	.081	-.092	[-0.405, 0.024]
Exposure to war	0.016	0.021	0.73	.467	.043	[-0.026, 0.058]
Depression						
ΔCTSR	0.102	0.011	9.53	<.001	.633	[0.081, 0.123]
CTSR T1	0.091	0.009	9.65	<.001	.674	[0.072, 0.109]
Age	0.001	0.004	0.35	.727	.020	[-0.006, 0.009]
Gender	0.008	0.129	0.06	.952	.003	[-0.245, 0.261]
Exposure to war	-0.017	0.025	-0.69	.488	-.043	[-0.066, 0.031]

Gender: 1 = Woman, 2 = Man. Anxiety *R*^2^ = .533. Depression *R*^2^ = .414. Fit indices: χ^2(15)^ = 38.09, *p* <.001; CFI = .972; RMSEA.

## 4 Discussion

The present study examined longitudinal change in CTSR during the Israel-Hamas war and its prospective associations with functioning, maladjustment, anxiety, and depressive symptoms. Using latent change score modeling, we found that CTSR increased substantially from the early phase of the war to a later phase several months afterward. Moreover, both baseline CTSR and within-person increases in CTSR were associated with greater functioning problems, maladjustment, anxiety, and depressive symptoms at follow-up. These associations were observed while controlling for age, gender, and war exposure, indicating that CTSR is prospectively associated with later outcomes beyond demographic characteristics and exposure level alone.

The central contribution of this study is that CTSR escalation over time was prospectively associated with later functional and psychological difficulties. This finding extends the emerging CTSR literature, which has largely focused on scale development, cross-cultural validation, and cross-sectional correlates. The original CTSR validation study established CTSR as a measurable construct and demonstrated its association with distress and functional impairment among Israeli adults exposed to ongoing security threats. Subsequent Ukrainian studies further supported the validity of CTSR in prolonged war contexts, while showing that its clinical expression may vary across civilian and military populations. The present study adds a crucial longitudinal layer by demonstrating that CTSR is not only a concurrent marker of distress but also a dynamic indicator of accumulating risk.

These findings are especially relevant because the Israel-Hamas war represents a prolonged and evolving threat environment in which civilians were exposed not only to the acute consequences of the October 7 attack but also to continuing missile alerts, uncertainty regarding hostages, military mobilization, displacement, media exposure, and concern about escalation on multiple fronts. Prior Israeli work conducted during weeks 4–6 of the war showed that CTSR was shaped by media exposure, forward-focused coping, and system justification, rather than direct exposure alone. The present findings suggest that as the war continued, CTSR remained clinically consequential not only as an early reaction to the prolonged exposure to adversity, but also as a process whose worsening predicted later impairment and affective symptoms.

Allostatic load theory provides a useful framework for interpreting this pattern. Allostasis refers to the process by which physiological systems maintain stability through adaptation to changing demands, whereas allostatic load reflects the cumulative cost of repeated or prolonged activation of stress-response systems (2, 13). When a threat is recurrent, unpredictable, and unresolved, neuroendocrine, autonomic, inflammatory, metabolic, and cognitive-affective regulatory systems may remain persistently engaged. Over time, this sustained mobilization can contribute to exhaustion, emotional dysregulation, reduced cognitive flexibility, sleep disturbance, and vulnerability to affective symptoms (1, 13).

Although the present study did not include biological markers of allostatic load, the increase in CTSR over time may be interpreted as a psychological manifestation of cumulative stress burden. CTSR includes symptoms that map conceptually onto the process of cumulative adaptation under threat: exhaustion and detachment may reflect depletion of regulatory resources; fear and helplessness may reflect sustained anticipatory threat; and anger or betrayal may reflect erosion of trust and perceived protection under prolonged insecurity (5). The fact that CTSR increased markedly from the early to the later phase of the war suggests that ongoing exposure may not simply maintain distress, but may intensify it as psychological resources become progressively strained.

Conservation of Resources theory offers a complementary explanation. According to Hobfoll’s Conservation of Resources theory, stress occurs when individuals experience actual or threatened resource loss, or insufficient resource gain, following substantial investment of coping effort (12). Prolonged war threatens multiple resources simultaneously, including perceived safety, predictability, family stability, daily routines, occupational functioning, social roles, trust in institutions, and the capacity to plan for the future. Individuals living under such conditions must continuously invest cognitive, emotional, and behavioral resources simply to maintain ordinary functioning. Over time, this can generate a loss spiral in which depleted resources increase vulnerability to further distress and impairment (12, 23).

From this perspective, CTSR escalation may reflect both allostatic burden and resource depletion. Allostatic load explains the cumulative physiological and affective cost of sustained threat mobilization, whereas the Conservation of Resources theory explains how prolonged exposure to adversity erodes the resources needed to cope, function, and adapt. Together, these frameworks provide a stronger theoretical basis for understanding why CTSR increased over time and why this increase is associated with later functioning problems, maladjustment, anxiety, and depression.

Our findings suggest that increased continuous stress responses may impair the capacity to adapt psychologically and functionally to an unstable environment. Furthermore, the prospective association of anxiety and depressive symptoms suggests that CTSR may operate as a transdiagnostic stress-response process. This interpretation is consistent with the Ukrainian civilian adaptation of the CTSR, which found positive associations with depression, anxiety, perceived stress, and PTSD symptoms, as well as negative associations with resilience. It is also consistent with the Ukrainian military adaptation, in which helplessness, depression, and psychosocial disruption emerged as core components of the CTSR profile. Thus, CTSR appears to capture a broader stress-response profile that may increase vulnerability to multiple forms of psychopathology.

Importantly, the role of war exposure across outcomes also deserves careful interpretation. In the current models, war exposure predicted functioning problems and maladjustment, but did not predict anxiety or depressive symptoms once CTSR and CTSR change were included. This pattern may indicate that objective exposure is more directly linked to practical disruption and adjustment demands, whereas affective psychopathology may be more strongly associated with the subjective cumulative response captured by CTSR. In other words, exposure may shape the external burden individuals face, while CTSR may capture the internal burden of carrying that threat over time. This interpretation is consistent with earlier Israeli findings showing that direct exposure did not independently associate with CTSR during the early phase of the war, whereas media exposure, coping, and system justification were more strongly associated with CTSR levels (11). However, it is worth noting that the current study did not capture the severity, duration, or subjective aspects of exposure, so its role is limited to measuring dichotomous objective exposure.

This study has several limitations that should be acknowledged. First and foremost, although the study used a longitudinal design, it included only two waves. This allowed us to examine change from the early to a later phase of the war, but it did not allow for modeling nonlinear trajectories or identifying heterogeneous longitudinal profiles, such as those identified in Bonnano’s (24) framwork who characterized trajectories by stable low distress or resilience, gradual recovery, chronic distress, and delayed or increasing difficulties. Future studies incorporating multiple assessment waves and person-centered trajectory analyses may clarify whether distinct patterns of CTSR emerge during prolonged conflict and which personal, social, and exposure-related factors differentiate these patterns. In addition, an important limitation is that depression, anxiety, adjustment difficulties, and functional impairment were not assessed at baseline. Consequently, the prospective analyses could not account for pre-existing differences in these outcomes or determine whether CTSR was associated with subsequent changes in emotional distress and functioning. The findings should therefore be interpreted as prospective associations rather than evidence that CTSR caused or independently predicted a deterioration in these aspects.

Second, the study relied on self-report measures, which are appropriate for assessing subjective stress responses, but they may be affected by response tendencies, and subjective interpretation of symptoms. Future research should incorporate clinician-administered assessments and behavioral indicators of functioning and, where possible, integrate biological and physiological markers, such as cortisol regulation, inflammatory markers, heart rate variability, sleep physiology, and blood pressure.

Third, attrition was substantial, although analyses indicated no significant baseline differences between responders and non-responders in CTSR, depression-anxiety, adjustment, or functioning. Nevertheless, attrition may still limit generalizability; combined with the predominantly female, Jewish sample characteristics, this limits the study’s conclusions to other demographic and cultural groups. It is worth noting that many civilian men were called to duty in the army, resulting in fewer men available to participate. Moreover, the sample may not represent minority groups or groups with more severe or direct exposure, such as bereaved families, relatives of kidnapped hostages, combat soldiers, and first responders. These populations may exhibit distinct CTSR trajectories, distinct risk and protective factors, and distinct relationships among exposure, CTSR, and later outcomes. Future research should examine mechanisms linking CTSR escalation to later impairment, including perceived threat, media exposure, sleep disturbance, coping strategies, social support, institutional trust, and moral or institutional injuries. Finally, given that CTSR includes emotional items such as helplessness and anger, it may partially share negative variance with affective outcomes (depression and anxiety). Future research may clarify the mutual relationships, as well as the shared and distinct variances.

The findings have several implications for clinical practice and public-health responses during prolonged conflict. First, they highlight the importance of evaluating continuous traumatic stress reactions along with standard measures of depression, anxiety, and adjustment difficulties, as these may not fully capture the experiences of those living under ongoing, active threat. CTSR assessment may help identify individuals whose distress reflects the cumulative burden of ongoing danger rather than only the aftermath of a discrete traumatic event. However, the present study cannot determine whether CTSR provides incremental explanatory or clinical value beyond pre-existing or concurrent psychological distress. Its incremental validity and clinical utility should therefore be examined in future research. Second, the findings highlight the potential importance of repeated assessment. A single assessment can identify individuals with high CTSR at a single time point, but it cannot detect escalation. The present study demonstrates that within-person increases in CTSR propectiviely associated with later functioning problems, maladjustment, anxiety, and depression. Nevertheless, because these outcomes were assessed only at the second wave, these findings cannot establish that increases in CTSR caused or independently predicted subsequent deterioration. In prolonged war contexts, monitoring changes in CTSR over time may therefore be clinically useful, particularly for detecting individuals whose distress is intensifying even if their exposure level has not changed substantially or they do not meet full PTSD criteria. Third, a CTSR-informed approach may help avoid overpathologizing adaptive responses to realistic danger while still identifying clinically significant deterioration. In an ongoing war, vigilance, fear, and avoidance may be contextually appropriate. The threshold for clinical concern may therefore be better indicated by escalation, exhaustion, maladjustment, impaired functioning, and affective symptoms. This distinction is essential in psychiatry and trauma care because diagnostic frameworks developed for post-event trauma may be less precise when danger remains ongoing. Finally, the findings suggest that public-health planning during prolonged conflict should not focus exclusively on acute trauma exposure. Prevention efforts should also address the accumulation and intensification of psychological burden over time.

Although the present study cannot establish CTSR as a distinct or independent intervention target, monitoring CTSR together with established indicators of mental health and functioning may contribute to identifying individuals and communities experiencing increasing distress. Because responses to prolonged conflict are shaped by personal, social, and contextual factors, effective prevention and intervention may require multi-level strategies targeting individuals, families, communities, and public systems.

Taking all together, the present study provides longitudinal evidence that CTSR increases over time during ongoing war and that both initial CTSR and escalation in CTSR prospectively associated with later functioning problems, maladjustment, anxiety, and depression. These findings support the conceptualization of CTSR as a dynamic response to continuous threat and as a clinically meaningful marker of cumulative psychological risk. By showing that within-person increases in CTSR prospectively associated with later impairment and affective symptoms, the study extends the CTSR literature beyond cross-sectional validation and highlights the importance of monitoring psychological responses over time in populations living under prolonged danger.

## Data Availability

The raw data supporting the conclusions of this article will be made available by the authors, without undue reservation.
